# *Chlorella sorokiniana* attenuates deoxynivalenol-exacerbated allergic dermatitis and type 2 immune responses in mice

**DOI:** 10.1007/s12550-026-00686-6

**Published:** 2026-10-16

**Authors:** Ayano Sato, Tetta Hiwatashi, Mao Kaneki, Chiharu Ohira, Minori Chiba, Kaho Keimatsu, Shiro Miyake, Taro Ogawa, Ayaka Nakashima, Tomoki Fukuyama

**Affiliations:** 1 Laboratory of Veterinary Pharmacology, School of Veterinary Medicine, Azabu University, 1-17-71 Fuchinobe, Chuo-Ku, Sagamihara, Kanagawa 252-5201 Japan; 2 Biosignal Research Center, Kobe University, Rokkodaicho 1-1, Nada-Ku, Kobe, Hyogo 657-8501 Japan; 3 Euglena Co., Ltd, 5-29-11 Shiba, Minato-Ku, Tokyo, 108-0014 Japan; 4 Center for Human and Animal Symbiosis Science, Azabu University, 1-17-71 Fuchinobe, Chuo-Ku, Sagamihara, Kanagawa 252-5201 Japan

**Keywords:** *Chlorella sorokiniana*; deoxynivalenol, Mycotoxin, Allergic dermatitis, Immunotoxicity, Type 2 inflammation

## Abstract

Chronic dietary exposure to low concentrations of deoxynivalenol (DON), a trichothecene mycotoxin found in cereal products, disrupts immune homeostasis and exacerbates allergic inflammation. *Chlorella sorokiniana* (*CS*) can adsorb DON and reduce its systemic absorption. However, whether this DON-binding activity protects against DON-induced immune dysregulation remains unclear. Here, we investigated the effects of *CS* on DON-induced toxicity and DON-exacerbated allergic dermatitis. In vitro, murine DC2.4 dendritic cells were exposed to DON in the presence or absence of *CS*-treated DON preparations. DON reduced cell viability and ATP-dependent luminescence while increasing IL-1β and TNFα production in a concentration-dependent manner. Pretreatment with *CS* attenuated these cytotoxic and pro-inflammatory effects. In vivo, chronic low-dose DON exposure aggravated toluene diisocyanate–induced allergic dermatitis, as demonstrated by increased ear swelling, scratching behavior, inflammatory cell infiltration, dermal edema, and cutaneous expression of *Il4*, *Il13*, and *Il33*. DON also increased dendritic, activated CD4^+^ T, and IgE^+^ B cell populations; type 2 cytokine production; and serum IgE levels. Oral *CS* administration attenuated local and systemic allergic responses. Contrastingly, *CS* exerted limited effects on allergic inflammation in mice not exposed to DON, indicating that its protective activity was primarily associated with the mitigation of DON toxicity rather than with nonspecific immunosuppression. Both conventional and pulverized *CS* reduced several DON-associated inflammatory responses. An in vitro binding assay confirmed that *CS* decreased residual DON concentrations. These findings demonstrate that *CS* reduces the bioavailability and immunotoxic effects of DON and may represent a functional dietary strategy for preventing the mycotoxin-associated exacerbation of allergic inflammation.

## Introduction

Mycotoxins are widely distributed contaminants in food and feed that exert toxic effects on vertebrates, even at low concentrations (Pestka 2010). Among these, deoxynivalenol (DON) is a representative trichothecene mycotoxin frequently detected in cereals and cereal-derived products, and chronic exposure through daily dietary intake has become an important toxicological concern. DON is relatively stable during food processing and may remain in contaminated commodities. Previous studies have shown that DON causes gastrointestinal injury, reduces feed intake, impairs epithelial barrier function, and disrupts immune homeostasis (Teng et al. 2026). In addition to these direct toxic effects, DON has been reported to enhance inflammatory responses and aggravate allergic inflammation, suggesting that its biological actions extend beyond tissue toxicity to include the modulation of immune responses (Payros et al. 2017; Seyed Toutounchi et al. 2021).

Current countermeasures against mycotoxin exposure primarily depend on monitoring contaminated raw materials and avoiding contaminated food or feed before ingestion. Although these approaches are essential for risk management, they do not eliminate exposure completely, particularly when low levels of contamination occur in commonly consumed food products. Once mycotoxins have been ingested orally, practical strategies for reducing their toxicological and immunological consequences remain limited. This limitation is particularly important for chronic exposure to low concentrations of mycotoxins, which may not cause acute toxicity but can disturb immune regulation and exacerbate inflammatory or allergic diseases (Yuen and Schoneweis 2007; Fumagalli et al. 2021; Soodamani et al. 2026). Therefore, functional materials that reduce mycotoxin absorption in the gastrointestinal tract may provide a useful complementary approach to prevent downstream immune abnormalities.

Recently, natural substances capable of adsorbing mycotoxins into the intestinal lumen and inhibiting their systemic absorption have attracted increasing attention. *Chlorella* species are freshwater microalgae that are widely consumed as food and dietary supplements because of their nutritional value and favorable safety profiles (Merchant and Andre 2001). Their cell wall components and abundant nutritional constituents may interact with various compounds in the digestive tract, making them promising candidates as toxin-binding materials. We previously reported that orally administered *Chlorella sorokiniana* (*CS*) cultivated in Japan binds to DON and ochratoxin A in vitro and markedly reduces their plasma and urinary concentrations in mice, indicating that *CS* substantially inhibits the systemic absorption of these mycotoxins (Yamaguchi et al. 2025). However, the present study mainly focused on the toxicokinetics of mycotoxin detoxification. Whether *CS*-mediated inhibition of DON absorption protects against DON-induced immune dysregulation or allergic inflammation remains unclear.

The immunological consequences of DON exposure are particularly relevant in allergic diseases. Low-dose or chronic oral exposure to DON has been suggested to disturb the immune balance, induce inflammatory cytokines, and enhance allergic responses (Aihara et al. 2020; Ookawara et al. 2021). In allergic contact dermatitis and related inflammatory skin conditions, antigen-presenting cells, activated T cells, IgE-producing B cells, and Th2-associated cytokines such as IL-4 and IL-13 contribute to the amplification of local and systemic inflammation. IL-33, an epithelial-derived alarmin, plays an important role in promoting type 2 immune responses and itch-associated skin inflammation. Thus, DON exposure may act as a dietary factor that worsens allergic inflammation by enhancing cytokine production and immune cell activation. Given these observations, whether reducing DON absorption in the gastrointestinal tract confers immunological protection remains to be determined. In the present study, we investigated whether *CS* attenuates DON-exacerbated allergic inflammation and suppresses DON-induced inflammatory activation. First, we used murine DC2.4 dendritic cells to evaluate whether *CS*-treated DON preparations reduce DON-induced cytotoxicity, impaired cellular metabolic activity, and production of inflammatory cytokines such as IL-1β and TNFα. Second, we used a mouse model of toluene diisocyanate (TDI)-induced allergic contact dermatitis to examine whether oral *CS* administration suppresses DON-enhanced ear swelling, scratching behavior, histological inflammation, Th2-related cytokine expression, lymph node (LN) immune activation, and serum IgE production. We also compared non-pulverized and pulverized *CS* preparations and assessed their ability to reduce residual DON concentrations. By integrating in vitro and in vivo analyses, we aimed to clarify the potential of *CS* not only as a DON-detoxifying material but also as a functional dietary strategy for preventing DON-associated aggravation of allergic inflammation.

## Materials and methods

### Cell culture and DON treatment in DC2.4 cells

The DON was provided by Dr. Shiro Miyake (Kobe University, Japan). The identity and concentration of DON were verified by UV spectrophotometry using the reported absorption maximum and molar absorptivity before use. Heated air–dried *CS* powder was obtained from Euglena Co., Ltd. (Tokyo, Japan). The *CS* powder used in this study contained 4.3 mg water, 69.58 mg protein, 5.1 mg ash, 0.098 mg sodium, 0.0273 mg iron, and 2.91 mg total chlorophyll per 100 mg. The murine dendritic cell line DC2.4 was obtained from the American Type Culture Collection (Manassas, VA, USA). Cells were maintained in Roswell Park Memorial Institute (RPMI)−1640 medium (FUJIFILM Wako Pure Chemical Corporation, Osaka, Japan) supplemented with 10% fetal calf serum (Sigma-Aldrich Co., LLC., Tokyo, Japan) and penicillin–streptomycin (FUJIFILM Wako Pure Chemical Corporation) under standard culture conditions. DC2.4 cells were seeded in 96-well plates at a density of 1 × 10^5^ cells/100 μL/well and cultured to approximately 70% confluence. To evaluate whether *CS* reduced DON-induced cytotoxicity and inflammatory activation, DON was prepared at 0, 0.1, 1, or 10 μg/mL in the presence or absence of *CS* pretreatment. For *CS* treatment, each concentration of DON was mixed with *CS* powder in ultrapure deionized water (100 mg/mL) and incubated for 10 min at room temperature. The mixture was centrifuged at 200 × *g* for 5 min, and the resulting supernatant was used for cell treatment.

### Assessment of cell viability, proliferation, and cytokine production

Cell viability was assessed using a Cytotoxicity LDH Assay Kit-WST (Dojindo Laboratories, Kumamoto, Japan). Briefly, 30 μL of culture supernatant was mixed with 30 μL of reaction solution and incubated for 30 min at room temperature in the dark. The reaction was terminated by adding 30 μL of 1 N HCl, and absorbance was measured at 490 nm using a microplate reader. Cellular proliferation/metabolic activity was evaluated using CellTiter-Glo 2.0 (Promega, Madison, WI, USA). The reagent was added to each well, mixed for 1 min, and ATP-dependent luminescence was measured using a luminometer. To assess inflammatory cytokine production, culture supernatants were collected after treatment, and IL-1β and TNFα concentrations were quantified using ELISA kits (R&D Systems, Minneapolis, MN, USA) according to the manufacturer’s instructions. Absorbance was measured at 450 nm using a Multiskan SkyHigh microplate reader (Thermo Fisher Scientific, Waltham, MA, USA).

### DON-binding assay using *CS *and pulverized *CS*

The DON-binding activity of *CS* was assessed by measuring the residual DON concentrations after incubation with *CS* preparations. The DON solution was mixed with *CS* or pulverized *CS* in ultrapure deionized water and incubated for 10 min at room temperature (100 mg/mL). After incubation, the mixture was centrifuged at 200 × *g* for 5 min, and the DON concentration in the supernatant was determined using an ELISA kit according to the manufacturer’s instructions (Nittobo Medical Co., Ltd., Tokyo, Japan). The detection limit of the DON kit was 4.12 ng/mL. The residual ratio was calculated as the ratio of the residual DON concentration in the supernatant to the initial DON concentration.

### Experimental animals

Seven-week-old female BALB/c mice were provided by Japan SLC, Inc. (Shizuoka, Japan). All mice were acclimated to the environment for at least 5 days and used for experiments at 8 weeks of age. A maximum of four mice were housed per cage under controlled conditions (lighting, 12 h light–dark cycle; temperature, 22 ± 2 °C; humidity, 55 ± 5%). The mice were provided with a certified pellet diet (CE-2, CLEA Japan, Inc., Tokyo, Japan) and tap water ad libitum. All the experiments were conducted in accordance with the Animal Care and Use Program of Azabu University (Approval No. 220316–46).

Mouse model of TDI-induced allergic dermatitis.

A mouse model of allergic dermatitis was established by repeated sensitization and challenge with toluene diisocyanate (TDI), as described previously by Ando et al. (Ando et al. 2023; Kishimoto et al. 2021), with minor modifications. Female BALB/c mice were used for the experiments. One day before the first sensitization, the abdominal hair was removed using a depilatory cream. On days 1–3, the abdominal skin was tape-stripped five times using adhesive tape, after which 5% TDI dissolved in acetone was topically applied to the exposed skin for sensitization. Three weeks after the initial sensitization, on day 22, the mice were re-sensitized by topical application of 0.5% TDI dissolved in acetone to enhance the allergic response. Seven days after re-sensitization, 0.5% TDI in acetone was applied to both sides of the ear auricles as the final challenge. The experimental schedule of TDI sensitization, DON exposure, and CS administration is shown in the corresponding figures. To evaluate the effect of chronic low-dose DON exposure on allergic inflammation, mice were exposed to DON at 0.3 μg/mL in drinking water throughout the experimental period. CS or pulverized CS was orally administered at 500 mg/kg body weight according to the experimental schedule. Control mice received the corresponding vehicle without CS. Drinking water and experimental treatments were provided continuously or at the indicated time points throughout the study. Immediately after the final TDI challenge, scratching behavior was recorded by video for 60 min. A scratching bout was defined as a series of scratching movements directed toward the challenged ear region and was counted as a single episode until the hind paw was returned to the floor or placed in the mouth. Ear thickness was measured immediately before and 24 h after the final TDI challenge using a thickness gauge, and the ear-swelling response was calculated as the difference between the pre-challenge and post-challenge measurements. At 24 h after the final challenge, the mice were euthanized under isoflurane anesthesia. Blood was collected for serum preparation, and the auricular lymph nodes and ear skin samples were subsequently harvested for further analyses. Ear tissues were used for histopathological examination and gene expression analysis, whereas auricular lymph nodes were used for flow cytometric analysis and cytokine release assays.

### Histological evaluation of ear skin

The ear skin samples were collected after the final TDI challenge and processed for histological evaluation. Sections were stained with hematoxylin and eosin and examined for inflammatory cell infiltration and edema. Histological changes were scored using a semi-quantitative scale as follows: 0, within normal limits; 1, mild; 2, moderate; and 3, severe. Results were expressed as the mean ± standard error of the mean (SEM). Histological scores were used to compare the effects of DON exposure and *CS* administration in the TDI-induced dermatitis model.

### Gene expression analysis in ear skin

Frozen ear skin samples were homogenized using a bead-beater homogenizer (μT-12; TAITEC Corporation, Saitama, Japan). Total RNA was extracted from the homogenized tissues using the NucleoSpin RNA kit (TaKaRa Bio Inc., Shiga, Japan) according to the manufacturer’s instructions. Subsequently, 120 ng of total RNA was reverse transcribed into complementary DNA using PrimeScript RT Master Mix (TaKaRa Bio Inc.). The expression levels of *Il4*, *Il13*, and *Il33* were quantified by real-time PCR using gene-specific primers (TaKaRa Bio Inc.), PowerUp SYBR Green Master Mix (Thermo Fisher Scientific), and CFX Duet Real-Time PCR System (Bio-Rad Laboratories Inc., Hercules, CA, USA). The thermal cycling conditions were set according to the manufacturer’s recommendations. Gene expression levels were normalized to *Actb* as the internal reference gene, and relative expression was calculated using the 2^−ΔΔCt^ method.

### Flow cytometric analysis of auricular LN immune cells

Single-cell suspensions were prepared from auricular LNs as described previously (Kaneki et al. 2023; Matsuzaka et al. 2025), and the total number of cells was determined using a CellDrop Cell Counting System (DeNovix Inc., Wilmington, DE, USA). To prevent nonspecific antibody binding, 1 × 10⁶ cells were preincubated with 1 μg of mouse Fc Block (Miltenyi Biotec K.K., Tokyo, Japan) before surface staining. The cells were subsequently incubated with fluorochrome-conjugated monoclonal antibodies against mouse CD3, CD4, CD11b, CD11c, CD19, CD44, CD62L, MHC class II, and IgE. Antibodies against CD3, CD4, CD11b, CD11c, CD19, CD44, CD62L, and MHC class II were obtained from BioLegend Inc. (San Diego, CA, USA) and Miltenyi Biotec K.K., whereas anti-mouse IgE antibody was obtained from Sony Biotechnology Inc. (Tokyo, Japan). DAPI was used to exclude nonviable cells. After staining, the cells were washed and analyzed using a BD FACSAria III cell sorter (BD Biosciences, Tokyo, Japan). Dendritic cell populations were identified as CD11c⁺ and CD11c⁺CD11b⁺ cells. Activated CD4⁺ T-cell subsets were defined as CD3⁺CD4⁺CD44⁺CD62L⁻ and CD3⁺CD4⁺CD44⁺CD62L⁺ cells. The IgE-bearing B cells were identified as CD19⁺IgE⁺ cells. The absolute number of each immune cell population was calculated from the total LN cell count, and the percentage of the corresponding population was determined using flow cytometry.

### Cytokine release assay of auricular LNs

Single-cell suspensions prepared from auricular LNs were used to evaluate cytokine production following T-cell stimulation. LN cells were seeded in 96-well plates at a density of 5 × 10^5^ cells/well and incubated with mouse T-activator CD3/CD28 Dynabeads (Thermo Fisher Scientific) for 24 h under standard culture conditions. After incubation, culture supernatants were collected and stored at − 80 °C until analysis. The concentrations of IL-4 and IL-13 in the culture supernatants were quantified using DuoSet ELISA kits (R&D Systems) according to the manufacturer’s instructions. Absorbance was measured at 450 nm using a microplate reader, and cytokine concentrations were calculated from standard curves generated using the corresponding recombinant cytokine standards.

### Measurement of total serum IgE concentration

Blood samples were collected from mice after the final TDI challenge, and serum was separated by centrifugation and stored at − 80 °C until analysis. The total serum IgE concentration was quantified using an ELISA kit (BioLegend Inc.) according to the manufacturer’s instructions. The absorbance was measured at 450 nm using a microplate reader, and the total IgE concentration was calculated from a standard curve generated using known IgE standards.

### Statistical analysis

All data are presented as the mean ± SEM. Statistical analyses were performed using GraphPad Prism, version 10 (GraphPad Software, San Diego, CA, USA). For in vitro experiments, multiple groups were compared using one-way analysis of variance (ANOVA), followed by Dunnett’s multiple-comparison test. For in vivo experiments, multiple-group comparisons were performed using one-way ANOVA, followed by Šídák’s multiple-comparison test. Histological scores were analyzed using the Mann–Whitney U test, with comparisons made against the TDI + DON group, where appropriate. Statistical significance was set at *p* < 0.05.

## Results

### *CS* attenuates DON-induced cytotoxicity and inflammatory cytokine production in DC2.4 cells

**Fig. 1:**
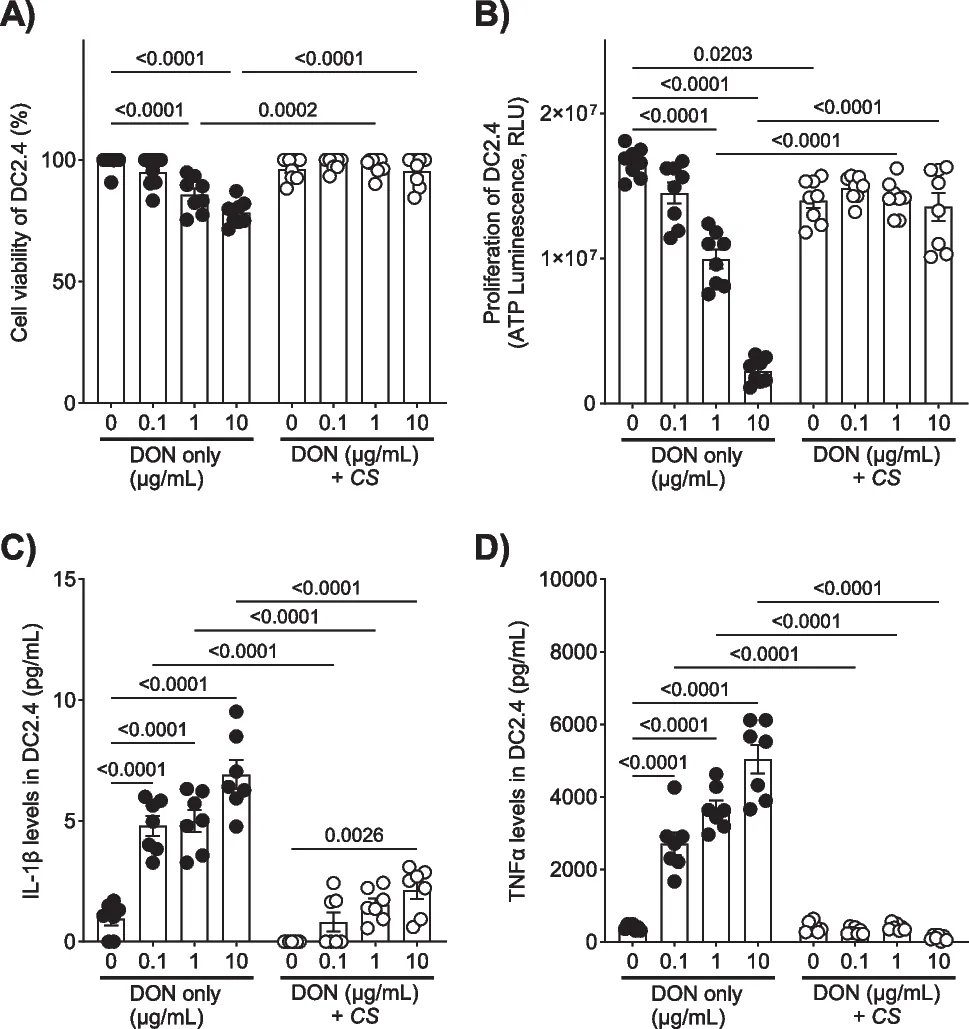
*Chlorella sorokiniana* (*CS*) suppresses deoxynivalenol (DON)-induced cytotoxicity and inflammatory cytokine production in DC2.4 cells. DC2.4 cells were exposed to DON (0, 0.1, 1, or 10 μg/mL) in the presence or absence of *CS*. DON and *CS* were preincubated in ultrapure water for 10 min and centrifuged prior to cell treatment. (**A**) DON reduced DC2.4 cell viability in a concentration-dependent manner, whereas *CS* co-treatment maintained cell viability close to control levels. (**B**) DON decreased ATP-dependent luminescence, indicating reduced cellular metabolic activity/proliferation; this reduction was attenuated by *CS* co-treatment. (**C**, **D**) DON markedly increased IL-1β and TNFα production in culture supernatants, whereas *CS* co-treatment suppressed these DON-induced cytokine responses. Data are presented as the mean ± standard error of the mean (SEM). Individual dots represent independent samples. Statistical analysis was performed using one-way analysis of variance (ANOVA) followed by Dunnett’s multiple comparison test. *p* < 0.05 was considered statistically significant. Significant differences between groups are indicated in the figure

To investigate whether *CS* modulates the toxic and inflammatory effects of DON, DC2.4 cells were exposed to DON at concentrations ranging from 0.1 to 10 μg/mL in the presence or absence of *CS*-treated DON preparations. As shown in Fig. 1A, exposure to DON significantly decreased DC2.4 cell viability in a concentration-dependent manner. The reduction in cell viability was particularly evident after exposure to DON at concentrations of 1 and 10 μg/mL. By contrast, pretreatment of DON with *CS* markedly attenuated the DON-induced loss of cell viability, maintaining viability at levels comparable to those of untreated control cells. Consistent with these findings, ATP-dependent luminescence, which reflects cellular metabolic activity and viable cell number, was significantly reduced after exposure to DON alone (Fig. 1B). The suppressive effect of DON on ATP-dependent luminescence increased with increasing DON concentration and was most pronounced at the highest concentration examined. Notably, *CS* treatment significantly restored ATP-dependent luminescence compared with the corresponding DON-alone groups, indicating that *CS* preserved cellular metabolic activity against DON-induced toxicity. To determine whether *CS* influenced DON-induced inflammatory responses, the production of pro-inflammatory cytokines was assessed. DON exposure considerably increased IL-1β secretion from DC2.4 cells in a concentration-dependent manner (Fig. 1C). Similarly, TNFα production was markedly elevated following DON treatment (Fig. 1D). In both assays, *CS* treatment significantly suppressed cytokine production compared with exposure to DON alone. The inhibitory effects of *CS* were particularly apparent at higher DON concentrations, at which IL-1β and TNFα production was reduced to levels close to those observed in untreated cells. These results demonstrate that *CS* attenuates both the cytotoxic and pro-inflammatory effects of DON in murine dendritic cells.

### Oral *CS* administration attenuates DON-exacerbated allergic skin inflammation

**Fig. 2:**
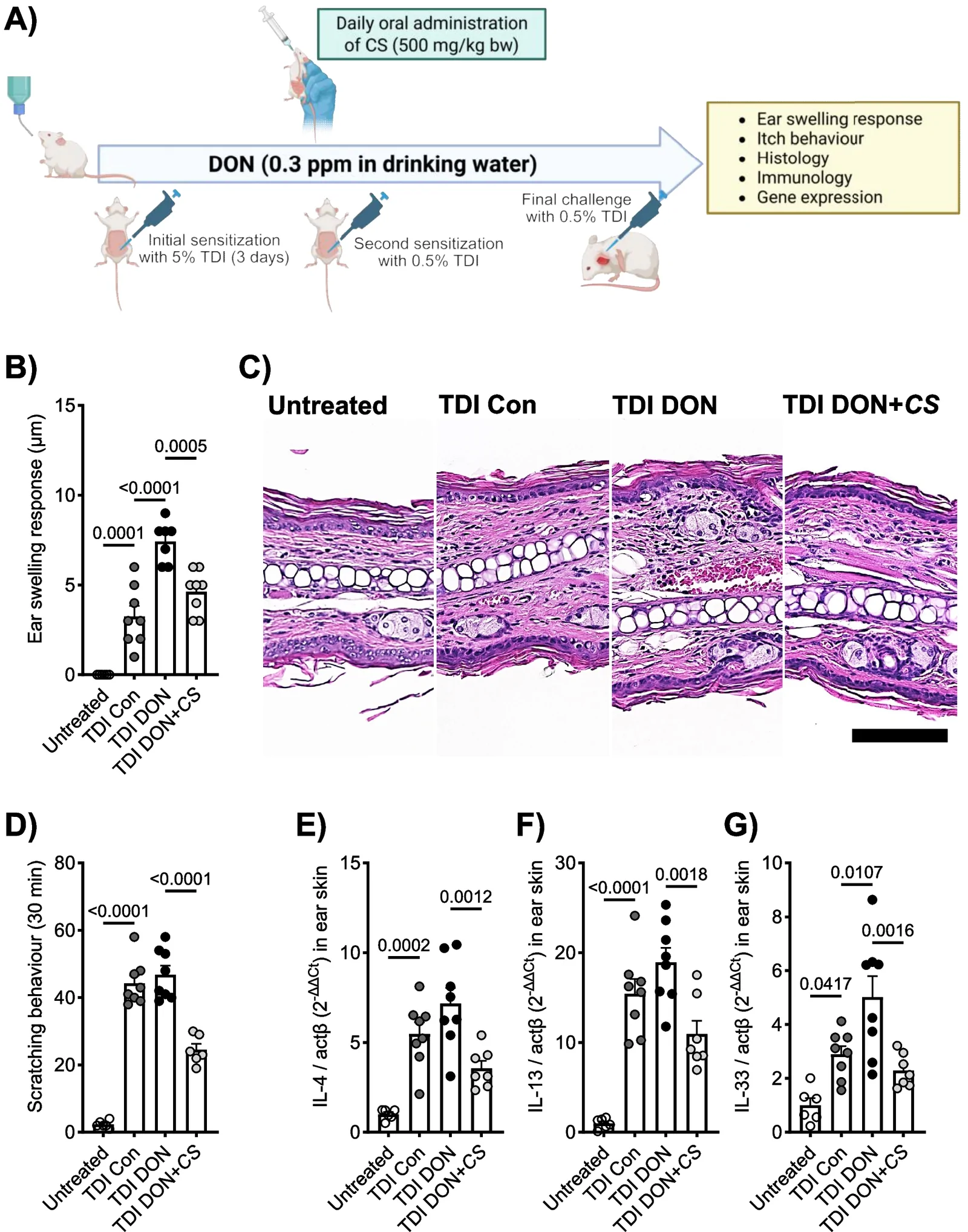
*Chlorella sorokiniana* (*CS*) attenuates deoxynivalenol (DON)-exacerbated allergic skin inflammation in a toluene diisocyanate (TDI)-induced mouse model. (**A**) Experimental protocol. Mice were exposed to DON in drinking water (0.3 µg/mL) and orally administered *CS* (500 mg/kg body weight [bw]) during the TDI-induced allergic dermatitis protocol. Ear swelling, scratching behavior, histology, immunological responses, and gene expression were evaluated after the final TDI challenge. (**B**) DON enhanced the TDI-induced ear swelling response, whereas *CS* administration reduced this exacerbation. (**C**) Representative histological images of ear skin show inflammatory changes induced by TDI and DON, which were attenuated by *CS* treatment. (**D**) DON increased scratching behavior after TDI challenge, and *CS* administration suppressed this increase. (**E**–**G**) DON enhanced the expression of Th2-associated inflammatory genes, including *Il4*, *Il13*, and *Il33*, in ear skin; these responses were reduced by *CS* treatment. Data are presented as the mean ± standard error of the mean (SEM). Individual dots represent independent samples. Statistical analysis was performed using one-way analysis of variance (ANOVA) followed by Šídák’s multiple comparisons test for comparisons among multiple groups. *p* < 0.05 was considered statistically significant. Significant differences between groups are indicated in the figure

To determine whether *CS* ameliorates DON-exacerbated allergic dermatitis in vivo, mice were exposed to DON in drinking water and orally administered *CS* during the induction of TDI-induced allergic contact dermatitis, according to the experimental schedule shown in Fig. 2A. TDI challenge induced a marked increase in ear swelling compared with that in untreated mice, and concomitant DON exposure further enhanced this response (Fig. 2B). Oral administration of *CS* significantly attenuated the DON-associated increase in ear swelling, indicating that *CS* reduces the aggravating effects of DON on TDI-induced skin inflammation. Histopathological examination of ear tissues revealed minimal inflammatory changes in the untreated mice, whereas TDI-treated mice exhibited inflammatory cell infiltration and dermal edema (Fig. 2C). These histopathological alterations were more pronounced in the TDI + DON group. By contrast, mice administered *CS* showed less inflammatory cell infiltration and edema than those exposed to TDI and DON without *CS* treatment. Consistent with the representative histological findings, semi-quantitative scoring demonstrated that both inflammatory cell infiltration and edema scores were increased by DON exposure and significantly reduced by *CS* administration (Table 1). DON exposure also enhanced scratching behavior following the TDI challenge (Fig. 2D). The number of scratching episodes was greater in the TDI + DON group than in the TDI-alone group, whereas oral *CS* administration significantly reduced the DON-associated increase in scratching behavior. The expression of genes associated with type 2 and epithelial inflammatory responses in ear skin was subsequently examined. TDI treatment increased the expression of *Il4*, *Il13*, and *Il33*, which was further enhanced by DON exposure (Fig. 2E–G). Oral *CS* administration significantly suppressed the DON-associated increases in the expression of these genes. Collectively, these findings indicate that *CS* attenuates the clinical, behavioral, histopathological, and molecular manifestations of allergic skin inflammation exacerbated by DON.

**Table 1:** Histological evaluation of the effects of *Chlorella sorokiniana* on deoxynivalenol-exacerbated ear inflammation in the TDI-induced allergic contact dermatitis mouse model

	**Untreated** **(n = 6)**	**TDI only** **(n = 8)**	**TDI + DON only** **(n = 8)**	**TDI + DON + *CS*** **(n = 8)**
Inflammatory cell infiltration	0.50 ± 0.22	1.00 ± 0.00	1.75 ± 0.25	1.00 ± 0.00 ^P = 0.0379^
Edema	0.00 ± 0.00	1.25 ± 0.16	1.88 ± 0.12	1.12 ± 0.12 ^P = 0.0104^

A histological score (0, within normal limits; 1, mild; 2, moderate; and 3, severe) was assigned to each observation. Results are expressed as the mean ± SEM. P values were calculated using the Mann–Whitney U test versus the TDI + DON group. *CS*, *Chlorella sorokiniana*; DON, deoxynivalenol; TDI, toluene diisocyanate

### *CS* suppresses DON-enhanced immune activation in auricular LNs

**Fig. 3:**
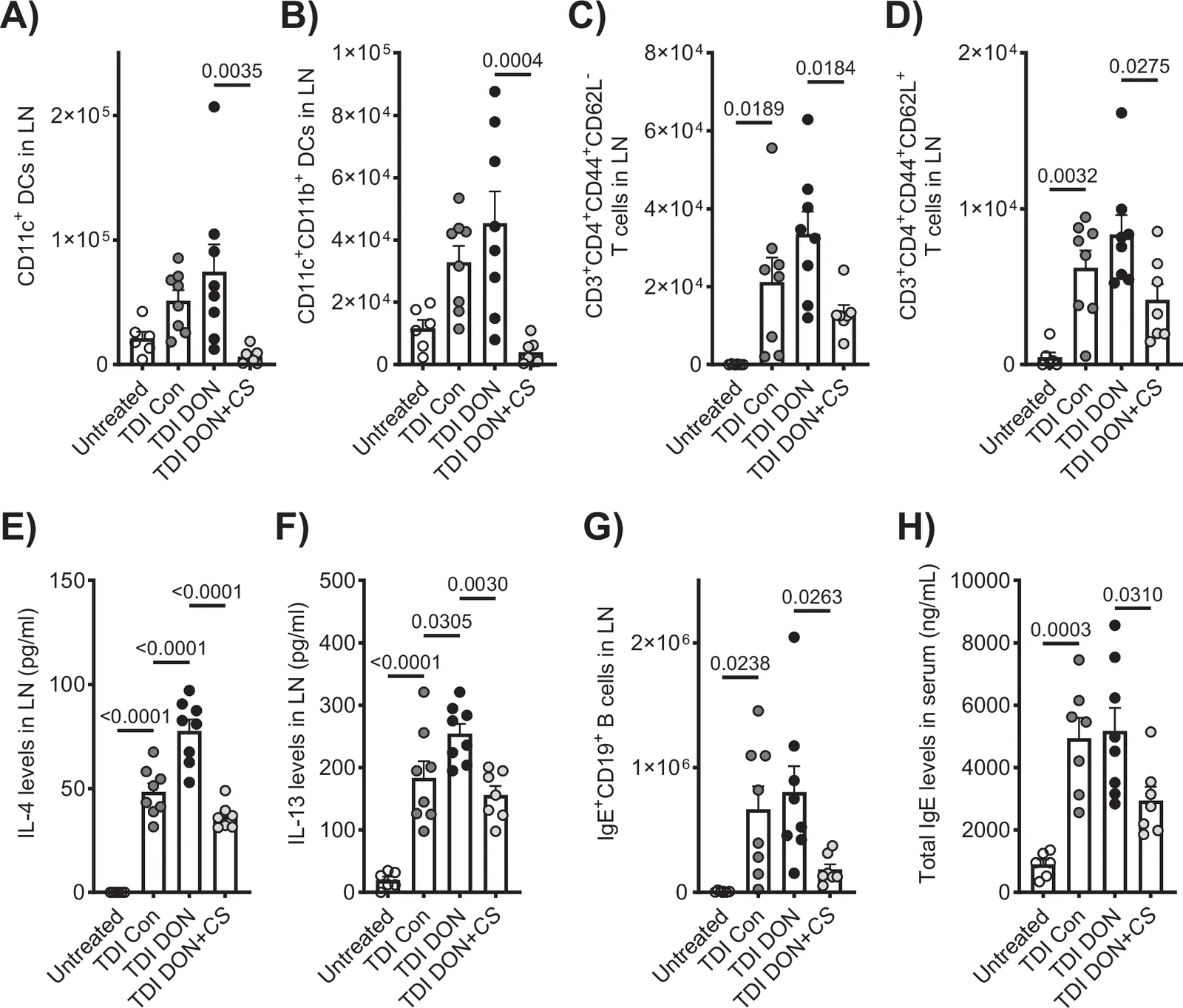
*Chlorella sorokiniana* (*CS*) suppresses deoxynivalenol (DON)-enhanced immune activation in lymph nodes (LNs) and serum IgE production in toluene diisocyanate (TDI)-treated mice. LN cells and serum were collected from mice subjected to TDI-induced allergic dermatitis with or without DON and *CS* treatment. (**A**, **B**) DON increased the numbers of CD11c^+^ and CD11c^+^CD11b^+^ dendritic cells (DCs) in LNs, whereas *CS* reduced these increases. (**C**, **D**) DON enhanced the accumulation of activated CD4^+^ T-cell subsets, including CD3^+^CD4^+^CD44^+^CD62L^−^ and CD3^+^CD4^+^CD44^+^CD62L^+^ T cells; these changes were attenuated by *CS*. (**E**, **F**) DON increased IL-4 and IL-13 levels in LNs, whereas *CS* suppressed these Th2 cytokine responses. (**G**) *CS* reduced the DON-associated increase in CD19^+^IgE^+^ B cell numbers in LNs. (H) DON-enhanced total serum IgE levels were reduced by *CS* treatment. Data are presented as the mean ± standard error of the mean (SEM). Individual dots represent independent samples. Statistical analysis was performed using one-way analysis of variance (ANOVA) followed by Šídák’s multiple comparisons test for comparisons among multiple groups. *p* < 0.05 was considered statistically significant. Significant differences between groups are indicated in the figure

To evaluate the effects of *CS* on regional immune responses, immune cell populations in auricular LNs were analyzed using flow cytometry. The number of CD11c^+^ dendritic cells increased following the TDI challenge and was further elevated in DON-exposed mice (Fig. 3A). Oral *CS* administration significantly reduced the number of CD11c^+^ cells compared with that in the TDI + DON group. A similar pattern was observed for the number of CD11c^+^CD11b^+^ dendritic cells, which increased following DON exposure and was significantly reduced after *CS* administration (Fig. 3B). DON exposure also enhanced the activation of CD4^+^ T cells. The numbers of CD3^+^CD4^+^CD44^+^CD62L^−^ and CD3^+^CD4^+^CD44^+^CD62L^+^ cells were higher in the TDI + DON group than in the TDI-alone group (Fig. 3C and D). *CS* administration significantly attenuated the DON-associated increases in both activated CD4^+^ T-cell subsets. To further characterize the type 2 immune response, the concentrations of IL-4 and IL-13 in the LN samples were measured. The levels of both cytokines increased following TDI challenge and were further elevated by DON exposure (Fig. 3E and F). Oral administration of *CS* significantly reduced IL-4 and IL-13 concentrations compared with those in the TDI + DON group. The number of CD19^+^IgE^+^ B cells also increased following DON exposure, whereas *CS* administration significantly reduced this population (Fig. 3G). Consistent with the LN findings, total serum IgE concentrations were elevated in the TDI + DON group and were significantly decreased by *CS* administration (Fig. 3H).

*CS* has limited effects on TDI-induced allergic inflammation in the absence of DON.

**Fig. 4:**
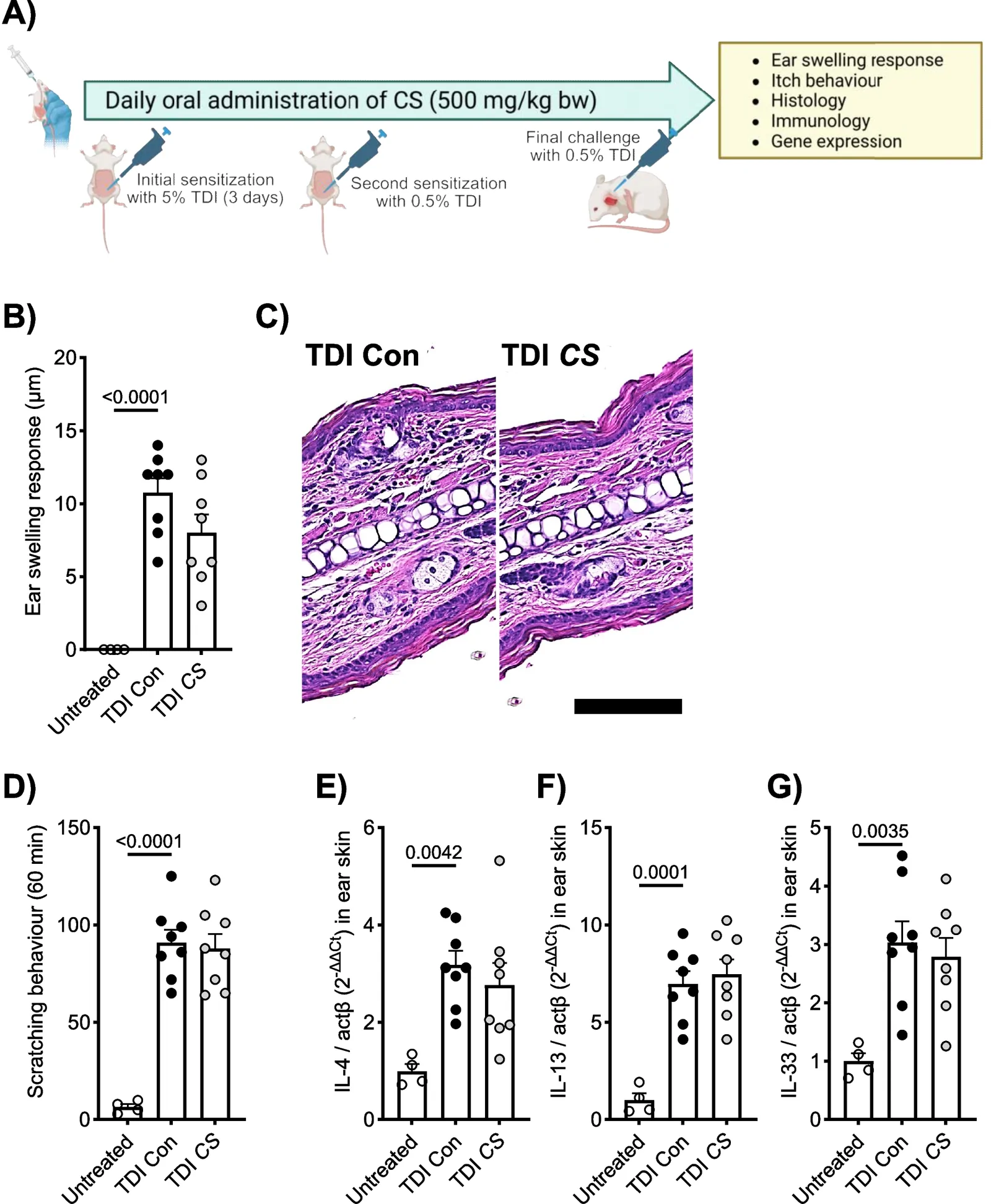
*Chlorella sorokiniana* (*CS*) alone does not markedly suppress toluene diisocyanate (TDI)-induced allergic skin inflammation in the absence of deoxynivalenol (DON). (**A**) Experimental protocol. Mice were orally administered *CS* (500 mg/kg body weight [bw]) during the TDI-induced allergic dermatitis protocol without DON exposure. (**B**) TDI induced ear swelling, and *CS* administration did not clearly suppress this response. (**C**) Representative histological images of ear skin from TDI control and TDI + *CS* groups. (**D**) TDI increased scratching behavior, which was not markedly reduced by *CS* alone. (**E**–**G**) TDI-induced expression of *Il4*, *Il13*, and *Il33* in ear skin was not substantially suppressed by *CS* administration in the absence of DON. Data are presented as the mean ± standard error of the mean (SEM). Individual dots represent independent samples. Statistical analysis was performed using one-way analysis of variance (ANOVA) followed by Šídák’s multiple comparisons test for comparisons among multiple groups. *p* < 0.05 was considered statistically significant. Significant differences between groups are indicated in the figure

**Fig. 5:**
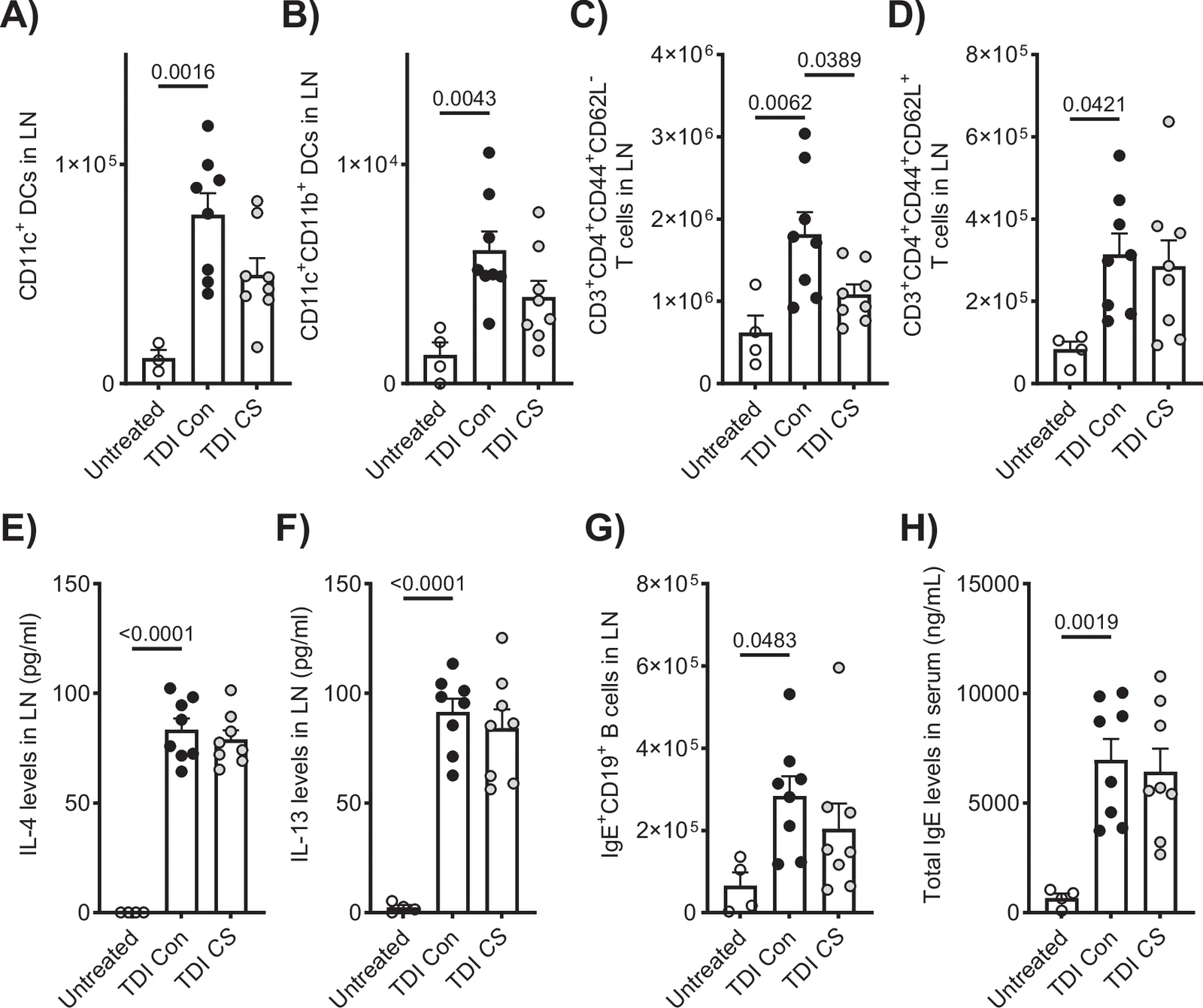
*Chlorella sorokiniana* (*CS*) alone does not markedly suppress toluene diisocyanate (TDI)-induced lymph node (LN) immune activation or serum IgE production in the absence of deoxynivalenol (DON). LN cells and serum were analyzed in mice subjected to TDI-induced allergic dermatitis with or without *CS* administration; however, without DON exposure. (**A**, **B**) TDI increased the numbers of CD11c^+^ and CD11c^+^CD11b^+^ dendritic cells (DCs) in LNs. (**C**, **D**) TDI increased the numbers of activated CD4^+^ T-cell subsets in LNs. (**E**, **F**) TDI elevated IL-4 and IL-13 levels in LNs. (**G**) CD19^+^IgE^+^ B cell numbers and (**H**) total serum IgE levels were increased by TDI treatment. *CS* administration alone did not clearly suppress these TDI-induced immunological responses. Data are presented as the mean ± standard error of the mean (SEM). Individual dots represent independent samples. Statistical analysis was performed using one-way analysis of variance (ANOVA) followed by Šídák’s multiple comparisons test for comparisons among multiple groups. *p* < 0.05 was considered statistically significant. Significant differences between groups are indicated in the figure

To determine whether *CS* directly suppresses allergic inflammation independent of its effects on DON, *CS* was administered to TDI-treated mice that were not exposed to DON, according to the experimental schedule shown in Fig. 4A. TDI challenge significantly increased ear swelling compared with that in untreated mice (Fig. 4B). However, *CS* administration did not significantly reduce the TDI-induced ear swelling response in the absence of DON. Histopathological examination revealed inflammatory cell infiltration and dermal edema in the ears of TDI-treated mice (Fig. 4C). The severity of these changes was comparable between the TDI-alone and TDI + *CS* groups. Consistent with these observations, no significant differences in inflammatory cell infiltration or edema scores were detected between the two groups (Table 2). TDI treatment also induced scratching behavior, whereas *CS* administration did not significantly alter the number of scratching episodes in mice that were not exposed to DON (Fig. 4D). Similarly, the expression levels of *Il4*, *Il13*, and *Il33* increased following the TDI challenge; however, *CS* administration did not consistently suppress these responses (Fig. 4E–G). Immune cell populations and cytokine responses in draining LNs were examined. TDI challenge increased the numbers of CD11c^+^ dendritic cells, CD11c^+^CD11b^+^ dendritic cells, activated CD4^+^ T-cell subsets, and CD19⁺IgE⁺ B cells (Fig. 5A–E). However, no marked reductions in these cell populations were observed following *CS* administration. Similarly, LN IL-4 and IL-13 concentrations and total serum IgE levels were not significantly altered by *CS* treatment in the absence of DON (Fig. 5F–H). These findings suggest that *CS* does not exert a strong general anti-allergic effect against TDI-induced dermatitis and that its protective activity is primarily associated with the attenuation of DON-mediated exacerbation.

**Table 2:** Histological evaluation of ear skin in the TDI-induced allergic contact dermatitis mouse model following *Chlorella sorokiniana* exposure

	**Untreated** **(n = 4)**	**TDI only** **(n = 8)**	**TDI + *CS* only** **(n = 8)**
Inflammatory cell infiltration	0.00 ± 0.00	1.65 ± 0.23	1.45 ± 0.20
Edema	0.00 ± 0.00	1.25 ± 0.16	1.00 ± 0.12

A histological score (0, within normal limits; 1, mild; 2, moderate; and 3, severe) was assigned to each observation. Results are expressed as the mean ± SEM. P values were calculated using the Mann–Whitney U test versus the TDI + DON group. *CS*, *Chlorella sorokiniana*; TDI, toluene diisocyanate

Conventional and pulverized *CS* attenuate DON-exacerbated allergic skin responses.

**Fig. 6:**
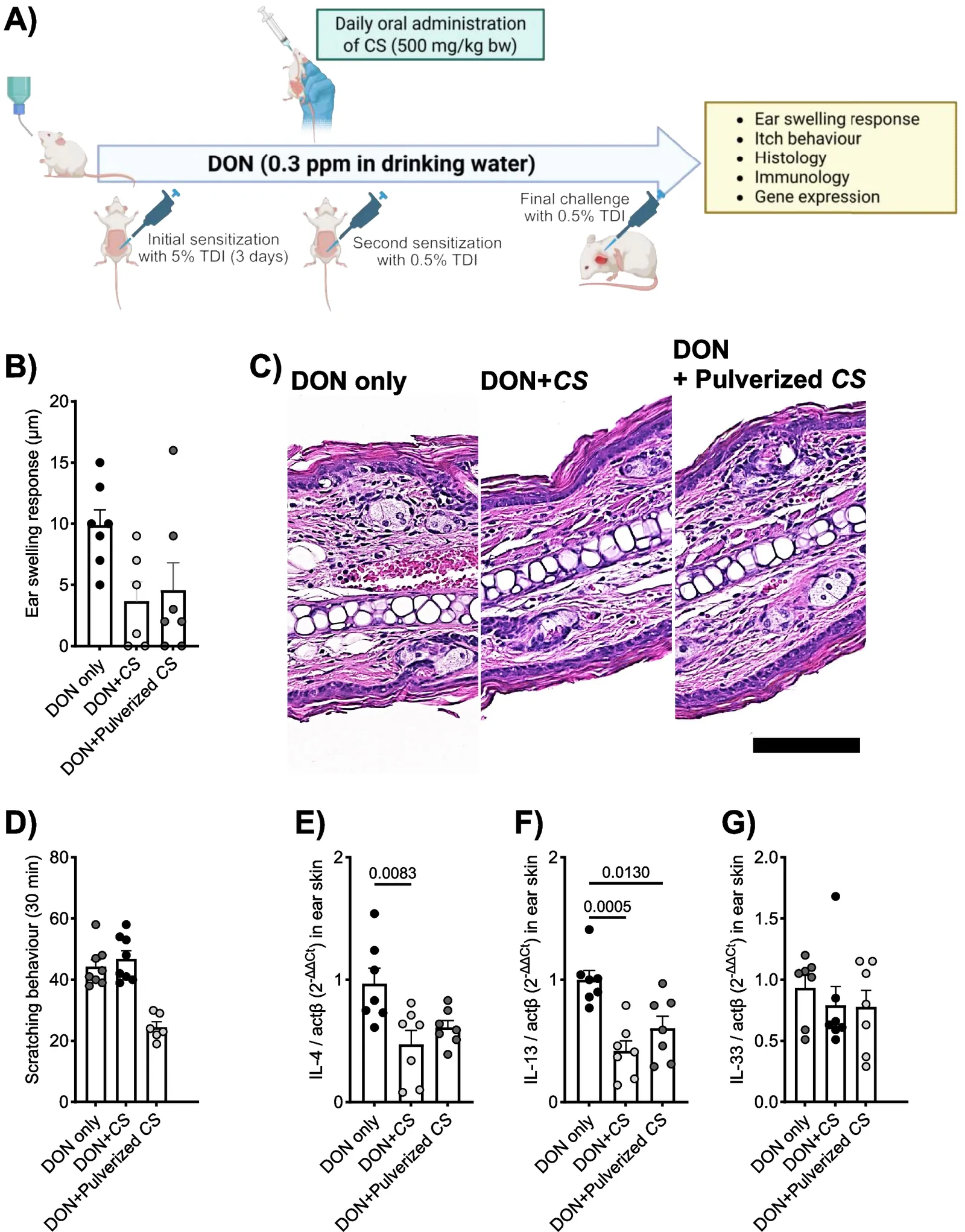
Pulverized *Chlorella sorokiniana* (*CS*) and non-pulverized *CS* attenuate deoxynivalenol (DON)-exacerbated allergic skin responses. (**A**) Experimental protocol. Mice were exposed to DON in drinking water (0.3 µg/mL) and orally administered *CS* or pulverized *CS* (500 mg/kg body weight [bw]) during the toluene diisocyanate (TDI)-induced allergic dermatitis protocol. (**B**) DON-induced enhancement of the ear swelling response was reduced by *CS* treatment, with comparable effects observed after pulverized *CS* administration. (**C**) Representative histological images of ear skin from DON-only, DON + *CS*, and DON + pulverized *CS* groups. (**D**) Scratching behavior was lower in the DON + pulverized *CS* group than in the DON-only group. (**E**–**G**) DON-associated increases in *Il4*, *Il13*, and *Il33* gene expression in ear skin were reduced by *CS* and/or pulverized *CS* treatment. Data are presented as the mean ± standard error of the mean (SEM). Individual dots represent independent samples. Statistical analysis was performed using one-way analysis of variance (ANOVA) followed by Šídák’s multiple comparisons test for comparisons among multiple groups. *p* < 0.05 was considered statistically significant. Significant differences between groups are indicated in the figure

**Fig. 7:**
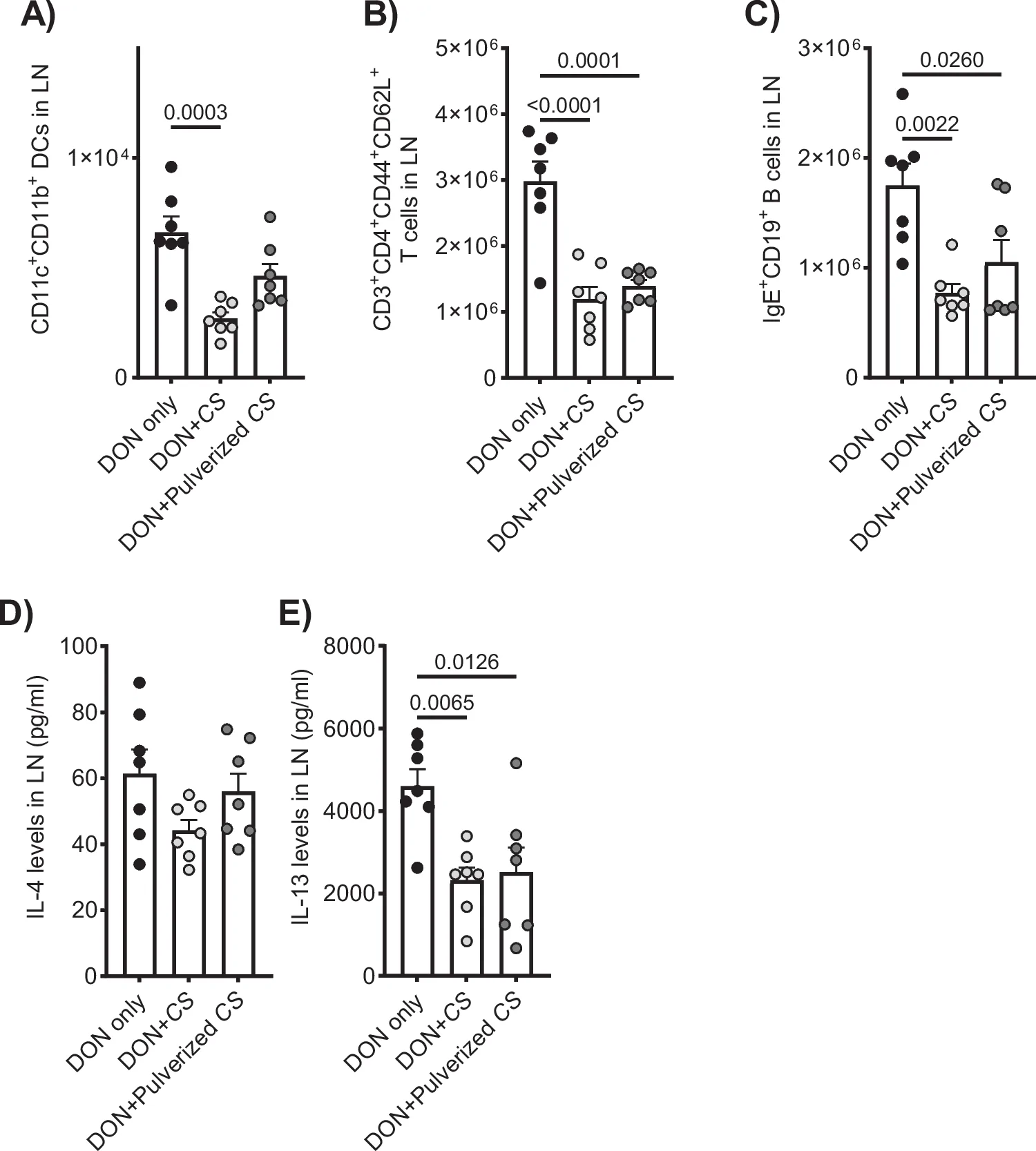
Pulverized *Chlorella sorokiniana* (*CS*) and non-pulverized *CS* suppress deoxynivalenol (DON)-associated immune activation in lymph nodes (LNs). LN cells and ear skin samples were analyzed in DON-exposed toluene diisocyanate (TDI)-treated mice administered *CS* or pulverized *CS*. (**A**) DON-associated increase in the number of CD11c^+^CD11b^+^ dendritic cells (DCs) in LNs was reduced by *CS* treatment. (**B**) Both *CS* and pulverized *CS* reduced the number of activated CD3^+^CD4^+^CD44^+^CD62L^+^ T cells. (**C**) *CS* reduced CD19^+^IgE^+^ B cell numbers in LNs, with a similar tendency observed after pulverized *CS* administration. (**D**, **E**) IL-4 and IL-13 levels in LNs were decreased by *CS* and/or pulverized *CS* treatment, particularly for IL-13. Data are presented as the mean ± standard error of the mean (SEM). Individual dots represent independent samples. Statistical analysis was performed using one-way analysis of variance (ANOVA) followed by Šídák’s multiple comparisons test for comparisons among multiple groups. *p* < 0.05 was considered statistically significant. Significant differences between groups are indicated in the figure

To compare the protective effects of conventional and pulverized *CS*, DON-exposed mice received either *CS* preparation orally during the induction of TDI-induced allergic dermatitis, according to the protocol shown in Fig. 6A. DON-exposed mice showed marked ear swelling following the TDI challenge. The administration of conventional or pulverized *CS* tended to reduce the ear swelling response, although the magnitude of the reduction differed between the two preparations (Fig. 6B). Histopathological examination revealed prominent inflammatory cell infiltration and dermal edema in the ears of DON-exposed mice (Fig. 6C). Both conventional and pulverized *CS* reduced the severity of these histological changes. Semi-quantitative analysis confirmed that the inflammatory cell infiltration score was significantly lower in both *CS*-treated groups than in the DON group (Table 3). The edema score was significantly reduced by both preparations, with a marked reduction observed following the administration of pulverized *CS*. Scratching behavior increased in DON-exposed mice following TDI challenge (Fig. 6D). Pulverized *CS* significantly reduced the number of scratching episodes, whereas conventional *CS* produced a similar but less pronounced reduction. The expression of *Il4*, *Il13*, and *Il33* in the ear skin was also assessed. DON exposure enhanced the expression of these inflammatory genes, whereas treatment with conventional or pulverized *CS* reduced these responses (Fig. 6E–G). In particular, *Il13* expression was significantly reduced following administration of pulverized *CS*. However, their effects on *Il4* and *Il33* expression were modest. The number of CD11c^+^CD11b^+^ dendritic cells in auricular LN increased in DON-exposed mice and was significantly reduced following the administration of conventional *CS* (Fig. 7A). Pulverized *CS* also exhibited a decreasing trend in cell population. The number of CD3^+^CD4^+^CD44^+^CD62L^+^ T cells was significantly reduced by both conventional and pulverized *CS* compared with that in the DON group (Fig. 7B). In addition, conventional *CS* significantly reduced the number of CD19^+^IgE^+^ B cells, and pulverized *CS* showed a similar trend (Fig. 7C). The LN IL-4 concentrations were not significantly different among the groups, although lower mean values were observed following *CS* administration (Fig. 7D). By contrast, IL-13 concentrations were significantly reduced by both conventional and pulverized *CS* compared with those in DON-exposed mice (Fig. 7E).

**Table 3:** Histological comparison of conventional and pulverized *Chlorella sorokiniana* in deoxynivalenol-exposed mice with TDI-induced allergic contact dermatitis

	**TDI + DON only** **(n = 7)**	**TDI + DON + *CS*** **(n = 7)**	**TDI + DON + Pulverized *CS*** **(n = 7)**
Inflammatory cell infiltration	1.71 ± 0.18	0.86 ± 0.26 ^P = 0.0311^	0.86 ± 0.14 ^P = 0.0088^
Edema	1.71 ± 0.18	1.00 ± 0.22 ^P = 0.0383^	0.43 ± 0.20 ^P = 0.0045^

A histological score (0, within normal limits; 1, mild; 2, moderate; and 3, severe) was assigned to each observation. Results are expressed as the mean ± SEM. P values were calculated using the Mann–Whitney U test versus the TDI + DON group. *CS*, *Chlorella sorokiniana*; DON, deoxynivalenol; TDI, toluene diisocyanate

### CS reduces the residual concentration of DON in vitro

**Fig. 8:**
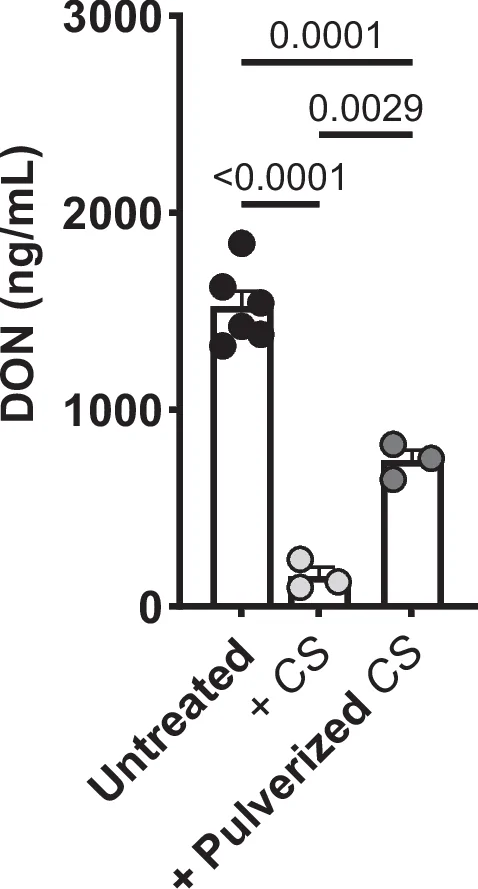
*Chlorella sorokiniana* (*CS*) and pulverized *CS* reduce deoxynivalenol (DON) levels in solution. DON concentrations were measured after incubation in the absence or presence of *CS* or pulverized *CS*. Both *CS* and pulverized *CS* significantly reduced detectable DON concentrations, indicating that both preparations retained DON-removing activity. Under the conditions of this assay, conventional *CS* reduced the detectable DON concentration to a greater extent than pulverized *CS*. Data are presented as the mean ± standard error of the mean (SEM). Individual dots represent independent samples. Statistical analysis was performed using one-way analysis of variance (ANOVA) followed by Šídák’s multiple comparisons test for comparisons among multiple groups. *p* < 0.05 was considered statistically significant. Significant differences between groups are indicated in the figure

To determine whether the protective effects of *CS* were associated with the direct binding or removal of DON, DON concentrations were measured after incubation with conventional or pulverized *CS*. High DON concentrations were detected in the untreated control solutions (Fig. 8). Incubation with conventional *CS* markedly reduced the residual DON concentration. Pulverized *CS* also significantly reduced the amount of detectable DON compared with that in the untreated solution. A significant difference in the residual DON concentration was observed between conventional and pulverized *CS* preparations. Under the conditions used in this assay, conventional *CS* reduced the detectable DON concentration to a greater extent than pulverized *CS*.

## Discussion

The present study demonstrates that the oral administration of *CS* attenuates allergic inflammation exacerbated by chronic low-dose DON exposure. Using both an in vitro dendritic cell system and an in vivo TDI-induced allergic contact dermatitis model, we showed that *CS* reduced the cytotoxic and pro-inflammatory effects of DON, suppressed the DON-associated aggravation of allergic skin lesions, and inhibited regional and systemic type 2 immune responses. In addition, *CS* markedly decreased the residual DON concentration in an in vitro binding assay. Collectively, these findings suggest that the protective effects of *CS* are attributable, at least in part, to its capacity to bind DON and reduce the levels of biologically available toxins.

DON is one of the most prevalent trichothecene mycotoxins in cereals and cereal-derived foods (Novak et al. 2018). Although exposure to high DON concentrations causes overt gastrointestinal toxicity, reduced food intake, and systemic illness, chronic exposure to lower concentrations may have subtle effects on immune homeostasis (Payros et al. 2017; Miao et al. 2025). Such exposure is particularly relevant to humans and animals because low-level contamination may occur continuously through daily food or feed intake. DON binds to ribosomes and activates stress-associated signaling pathways, resulting in altered protein synthesis, cellular injury, and inflammatory cytokine production. Depending on the concentration, exposure duration, and target cell type, DON can induce immune activation or immunosuppression. The present results support the concept that low-dose DON exposure can enhance inflammatory and allergic immune responses rather than simply causing nonspecific toxicity.

In DC2.4 cells, DON caused a concentration-dependent reduction in cell viability and ATP-dependent luminescence. The reduction in ATP-dependent luminescence likely reflects impaired cellular metabolic activity, reduced viable cell numbers, or both. Dendritic cells are highly sensitive to environmental stressors because their activation, survival, and antigen-presenting functions are closely linked to changes in cellular metabolism (Zhang et al. 2026; Acosta-Ampudia et al. 2026). Therefore, DON-mediated injury to dendritic cells may substantially alter the subsequent immune responses. Importantly, pretreatment of DON with *CS* preserved both cell viability and ATP-dependent luminescence, indicating that *CS* directly reduced the toxic activity of DON. DON also markedly increased IL-1β and TNFα production by DC2.4 cells. These cytokines are central mediators of innate immune activation and can promote the recruitment and activation of other immune cells (Kundu et al. 2026). TNFα contributes to tissue inflammation and barrier disruption, whereas IL-1β amplifies inflammatory signaling and may enhance T-cell activation. The increased production of these cytokines by antigen-presenting cells may create an inflammatory environment that favors the development or exacerbation of allergic responses (Feng et al. 2026). *CS* significantly reduced DON-induced IL-1β and TNFα production, particularly at the higher DON concentrations. Because the cells were treated with *CS*-treated DON preparations rather than with *CS* alone, this result strongly suggests that *CS* decreased the effective concentration or biological availability of DON in the culture medium.

These in vivo findings further demonstrate that DON aggravated TDI-induced allergic skin inflammation. Compared with TDI treatment alone, concomitant DON exposure increased ear swelling, scratching behavior, inflammatory cell infiltration, dermal edema, and the expression of *Il4*, *Il13*, and *Il33* in ear tissue. These changes indicate that DON enhanced several distinct components of allergic dermatitis, including vascular and tissue edema, inflammatory cell recruitment, pruritic responses, and type 2 cytokine signaling. IL-4 and IL-13 are key cytokines involved in type 2 immune responses (Zhang et al. 2026). IL-4 promotes the differentiation of naïve CD4^+^ T cells toward a Th2 phenotype and supports immunoglobulin class switching to IgE (Baumann and Roesner 2026). IL-13 contributes to tissue inflammation, epithelial dysfunction, and allergic disease severity (Feng et al. 2026; Zhu et al. 2026). In the skin, these cytokines impair barrier-related functions and promote chronic inflammatory responses (Baumann and Roesner 2026). IL-33 is an epithelial-derived alarmin that is released following tissue damage or cellular stress. It activates group 2 innate lymphoid cells and other immune populations, thereby amplifying type 2 inflammation (Nagashima et al. 2022). The increased expression of these genes in DON-exposed mice suggests that DON specifically amplifies pathways associated with allergic and type 2 immunity rather than merely increasing nonspecific inflammation. Oral *CS* administration significantly attenuated these DON-associated changes. The reduction in ear swelling and scratching behavior indicate that *CS* improved both objective inflammatory severity and itch-associated behavioral responses. Histopathological analysis revealed that *CS* reduced inflammatory cell infiltration and edema. These histological findings are important because they confirm that improvements in external and behavioral parameters were accompanied by reduced tissue inflammation. Moreover, the suppression of *Il4*, *Il13*, and *Il33* expression indicates that *CS* reduced the molecular signals underlying the allergic response.

The analysis of auricular LNs provided further insights into the immunological effects of DON and *CS*. DON treatment increased the number of CD11c^+^ and CD11c^+^CD11b^+^ dendritic cells. These populations are involved in antigen uptake, migration, and presentation to the T cells. An increase in the number of dendritic cells in the LNs may enhance the efficiency of allergen presentation and promote the expansion of allergen-reactive T cells. *CS* administration significantly reduced these dendritic cell populations, suggesting that it suppressed the DON-associated enhancement of antigen-presenting cell activation or accumulation. DON exposure also increased the number of activated CD4^+^ T-cell subsets. This observation is consistent with enhanced antigen presentation and amplification of adaptive immune responses. Simultaneously, IL-4 and IL-13 concentrations in the LNs increased, demonstrating that the expanded or activated T-cell response was accompanied by enhanced type 2 cytokine production. *CS* reduced the number of activated CD4^+^ T-cell subsets and lowered LN IL-4 and IL-13 concentrations. These results suggest that *CS* inhibited the progression of DON-associated innate immune activation to downstream type 2 adaptive immunity.

Humoral immune response was also affected by DON. The increase in the number of CD19^+^IgE^+^ B cells and total serum IgE levels indicates that DON enhanced the B-cell component of the allergic response. IgE plays a central role in immediate hypersensitivity and can amplify allergic inflammation through mast cell and basophil activation (Bubnoff et al. 2003). Although TDI-induced contact dermatitis is not exclusively dependent on IgE, increased IgE production reflects broader type 2 immune activation. The reduction in the number of IgE^+^ B cells and serum IgE levels following *CS* administration further supports the conclusion that *CS* suppresses systemic allergic immune activation associated with DON exposure.

A particularly important observation was that *CS* had a limited effect on TDI-induced dermatitis in the absence of DON. *CS* administration alone did not consistently reduce ear swelling, scratching behavior, histopathological inflammation, skin cytokine expression, LN immune cell activation, or serum IgE levels in mice that were not exposed to DON. This finding argues against the strong, nonspecific immunosuppressive or broad anti-allergic effects of *CS* under the conditions used in this study. Instead, the beneficial effects of *CS* were most apparent in the presence of DON. This distinction supports the hypothesis that *CS* primarily acts by interfering with DON exposure rather than directly suppressing the immune system.

The DON-binding assay provided a plausible mechanistic explanation for these findings. Incubation with *CS* markedly reduced the residual DON concentration in the supernatant. This result is consistent with previous observations that *CS* adsorbs DON and reduces its systemic absorption. The cell wall and surface components of *CS* may provide multiple binding sites for DON via hydrophobic interactions, hydrogen bonding, electrostatic interactions, or physical adsorption. Proteins, polysaccharides, pigments, and minerals may also contribute to toxin binding. By decreasing the free DON concentration in the gastrointestinal lumen, *CS* may reduce intestinal absorption and consequently limit the systemic exposure of immune tissues to DON. The effects of conventional and pulverized *CS* were also compared. Both preparations attenuated several manifestations of DON-exacerbated allergic inflammation, including histological inflammation, activated immune cell populations, and IL-13-related responses. Pulverized *CS* had pronounced effects on certain endpoints, including edema and scratching. However, the in vitro DON-removing assay indicated that conventional *CS* produced a greater reduction in detectable DON than pulverized *CS* under the experimental conditions. Therefore, the biological effects of pulverization may not be explained solely by the increased DON-binding capacity.

Pulverization can alter particle size, surface properties, dispersibility, gastrointestinal transit, and accessibility of intracellular components. Smaller particles may be distributed more evenly in the gastrointestinal tract or interact differently with the intestinal contents. Pulverization may also release bioactive components from *CS* that have immunological or barrier-supporting activities independent of direct binding to DON. Conversely, excessive disruption of the cell structure may alter or reduce the number of surface binding sites. These possibilities may explain why the relative effects of conventional and pulverized *CS* differed in the binding assays, histological outcomes, and immune parameters. Further physicochemical characterization of both preparations is required to clarify these differences.

Although the present study supports the DON-binding mechanism, additional mechanisms cannot be excluded. *Chlorella* contains proteins, polysaccharides, chlorophylls, carotenoids, vitamins, and other compounds that may influence oxidative stress, intestinal barrier integrity, and immune responses (Lousada Falcao et al. 2023). *CS* may therefore provide protection not only by binding to DON but also by reducing DON-induced oxidative or inflammatory stress. However, the limited effect of *CS* on mice not exposed to DON suggests that a direct immunomodulatory effect is insufficient to substantially suppress TDI-induced dermatitis. Thus, toxin removal appears to be the predominant mechanism in this experimental setting.

This study has some limitations. First, DON concentrations in plasma, urine, feces, intestinal contents, and tissues were not measured in the allergic dermatitis experiments. Direct toxicokinetic data can strengthen the relationship among *CS* administration, reduced DON absorption, and improved immune outcomes. Second, the binding assay was conducted under simplified in vitro conditions. The gastrointestinal environment has variable pH, digestive enzymes, bile acids, nutrients, and competing compounds that may influence DON adsorption. Therefore, simulated gastrointestinal digestion and in vivo recovery experiments were required. Third, this study focused primarily on type 2 immune responses and selected dendritic, T, and B cell populations. Other immune pathways, including regulatory T cells, innate lymphoid cells, mast cells, eosinophils, and Th17-associated responses, may also contribute to DON-exacerbated allergic inflammation. Fourth, the effects of *CS* on the intestinal barrier integrity and gut microbiota were not evaluated. As DON can disrupt the intestinal epithelium and alter microbial communities, *CS*-mediated protection of the gut environment may indirectly influence systemic immune responses and skin inflammation. Finally, the TDI-induced dermatitis model reflects chemically induced allergic inflammation and does not fully reproduce all aspects of naturally occurring atopic dermatitis. Confirmation in additional models, including house dust mite–induced dermatitis and spontaneous atopic dermatitis, would improve the generalizability of these findings. Studies on livestock or companion animals chronically exposed to contaminated feed would also be valuable because dietary mycotoxin exposure is an important veterinary concern.

In conclusion, we demonstrated that *CS* attenuates DON-induced cytotoxicity and inflammatory cytokine production in dendritic cells and reduces DON-exacerbated allergic dermatitis in mice. Oral *CS* administration suppressed skin inflammation, scratching behavior, type 2 immune responses, dendritic cell accumulation, activated CD4^+^ T-cell responses, IgE^+^ B-cell numbers, and serum IgE levels. The limited efficacy of *CS* in the absence of DON and its ability to reduce residual DON concentrations supports a mechanism based primarily on toxin binding and reduced bioavailability. These findings identify *CS* as a promising dietary supplement for mitigating the immunotoxic and allergy-promoting effects of chronic exposure to low doses of DON.

## Data Availability

No datasets were generated or analysed during the current study.

## Abbreviations

*CS*: *Chlorella* *sorokiniana*
DON: Deoxynivalenol
LN: Lymph node
TDI: Toluene diisocyanate
